# Gender differences in the use of transportation services to community rehabilitation programs

**DOI:** 10.1186/1471-2318-9-24

**Published:** 2009-06-27

**Authors:** Nanako Tamiya, Li-Mei Chen, Yasuki Kobayashi, Mariko Kaneda, Eiji Yano

**Affiliations:** 1 Department of Health Services Research, Graduate School of Comprehensive Human Sciences, University of Tsukuba, 1-1-1 Tenno-Dai, Tsukuba-Shi, Ibaraki, 305-8575, Japan; 2 School of Human Welfare Studies, Kwansei Gakuin University, 1-155 Ichiban-Cho, Uegahara, Nishinomiya-Shi, Hyogo, 662-8501, Japan; 3 Department of Public Health, University of Tokyo, 7-3-1 Hongo, Bunkyo-Ku, Tokyo, 113-0033, Japan; 4 Arakawa City Office, Tokyo Metropolitan Government, 2-2-3 Arakawa, Arakawa-ku Tokyo, 116-8501, Japan; 5 Department of Public Health, School of Medicine, Teikyo University, 2-11-1 Kaga, Itabashi-Ku, Tokyo, 173-8605, Japan

## Abstract

**Background:**

Prevention and reduction of disability among community-dwelling older adults have been an important health policy concern in Japan. Moreover, it has also become a gendered issue due to the recent rapid growth in older females than males with disability living in their own homes. The aim of this study is to examine whether there is a gender difference in the use of community rehabilitation programs in Japan, and if so, whether the lack of transportation services and accompanying caregivers are the reasons for the gender difference.

**Methods:**

This study was based on surveys of the program administrators and the primary caregivers of the program participants from 55 randomly selected community rehabilitation programs (CRP) in the Tokyo metropolitan area. Questions included sociodemographic characteristics of program participants, types of transportation services provided by the CRP, caregiver's relationship to participant, and the nature of family support. Bivariate statistical analysis was conducted.

**Results:**

Although there were more females than males with disability residing in communities, our findings showed that females were less likely to use CRP than males (1.3% and 2.3%, respectively; *X*^2 ^= 93.0, p < 0.0001). Lower CRP use by females was related to lower availability of transportation services (36% without transportation service and 46% door-to-door services) and fewer caregivers accompanying the participants to CRP.

**Conclusion:**

This study builds on previous research findings, which suggest gender inequality in access to CRP.

## Background

**Figure 1:**
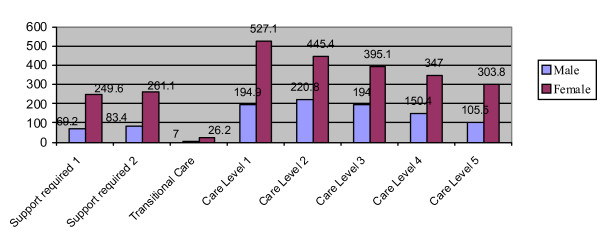
**Number of long-term care insurance enrollees by levels of care, year 2007 (1,000 per unit)**.

Prevention and reduction of disability among community-dwelling older adults have been an important health policy concern in Japan. It has also become a gendered issue especially with the recent rapid growth of females than males with disabilities. According to the Annual Report on the Aging Society, 4,175,000 or 16.6% of the total population age 65 years and older in 2005 were persons with disabilities. Approximately 73 percent of older adults with disabilities eligible for care under the national long-term care insurance were females [1]. More females than males reported difficulty with performing daily activities. In particular, "going outside" (11.7%) was identified as the most difficult activity for older females (Figure 1) [1].

In response to rehabilitating post-hospital discharge patients back to their communities, community-based rehabilitation programs (*Kinō Kunren Jigyō*, hereinafter CRP) were implemented in municipalities nationwide as a government-led initiative beginning in 1983. These programs offered patients, regardless of their disease types, the following services: health checkup; rehabilitation for walking, upper body functioning or activities of daily living; and limited recreational activities all for approximately two hours per visit (twice a week), basically for 6 months. In 1996, a second type of CRP was created to offer once a week rehabilitation and prevention from social withdrawal or loneliness due to older adults' declining physical and mental health basically for 12 months. Then, the implementation of the national long-term care insurance (NLTCI) in the year 2000 created various health and personal social services for older adults by combining previous social and health services under a different law [2]. CRPs were included under the NLTCI as one of the home care services ("day care with rehabilitation"). On the other hand, social day care, which existed as one of the social services under the previous law, provided recreation and social activities (not rehabilitation) under the NLTCI as "day service." However, despite the growth of unique CRPs under NLTCI, supply has not met the growing demand especially for home rehabilitation programs. Statistics show that approximately 47,600 beneficiaries were using home rehabilitation program while approximately 364,400 beneficiaries were using CRPs [3].

Although many health and social welfare services like CRPs were established to improve older adults' health, there are many structural barriers within the system which require necessary improvements. In particular, transportation has been identified as an important means for realizing needed access to health care and social services for older adults. In the year 2000, the Japanese government passed the "Law for Promoting Infrastructure for Accessible Transportation to Older Adults and Persons with Disabilities" targeting for improvements in the transportation system necessary for normalization [4]. However, despite the enactment of such law, a recent government study reported a significant number of older adults and persons with disabilities still experienced difficulties with transportation. For example, approximately 40.8% of persons with disabilities reported difficulty when accessing hospital and medical services [5].

A review of few existing studies examining the role of transportation for accessing health care revealed inconsistent findings on service use by gender. A study on the use of preventive services in the United States showed that females were significantly more likely than males to report a lack of transportation or distance as their main reason for not getting needed medical care (11% vs. 6%) [6]. Barusch and Spaid's study which investigated the use of home care services also reported findings that were leaning towards more females using transportation services than men [7].

As for CRP use, gender differences have been reported in other countries, too [8,9]. Our previous research with a small sample suggested that there were fewer females using CRP despite the fact that a number of older females with disabilities have surpassed older males [10]. However, there is no other research to date confirming gender inequality in CRP use in Japan. More empirical evidence is warranted on this issue.

In sum, a study examining the role of transportation is called for due to limited evidence confirming the importance of transportation in accessing health care for older adults coupled with the pressing need for adequate and efficient health policies amid budgetary constraints and increasingly aging population. Moreover, it has been reported that the most vulnerable, at-risk older adults were found to underutilize health services largely due to the unavailability of transportation [7]. Our previous study [10] suggested that the lower use of female may be due to the lack of transportation services and accompanying caregivers. As far as we know, no other study examined the gender difference of accessibility and use of transportation.

Hence, the aim of this study is to examine whether there is a gender difference in the use of community rehabilitation programs in Japan with estimated utilization rate of CRPs of relatively broad Tokyo metropolitan area, and if so, whether the lack of transportation services and accompanying caregivers are the reasons for the gender differences. Our research question is to test the hypotheses reported in our previous study as follows: (1) Is there lower CRP use by Japanese females than males, and if so, (2) Is lower CRP use by Japanese women due to the lack of comprehensive transportation services and accompanying family members to the programs? Implications for health policy and future research will also be provided.

## Methods

This study was conducted as a joint study with the Tokyo Metropolitan Government in 1994 prior to the addition of a second type of CRPs offering social activities for older adults in the year 1996. It was also prior to the implementation of the national long-term care insurance in the year 2000. Therefore, community rehabilitation programs (CRP) were designed under the Public Health Law for Older Persons (*rojin hoken ho*), and were administered by local municipalities with the objectives of rehabilitating older persons with functional disabilities who were discharged from hospitals. Hence, we conducted our study on the basis that these rehabilitation programs were similar, but differed in the provisions of transportation for accessing these programs.

Among all community rehabilitation programs (CRP) in the Tokyo metropolitan area (N = 220), one-fourth of the programs were randomly selected from a list of programs prepared by the Tokyo Metropolitan Government for this study. All of these programs were administered by municipalities under the Tokyo Metropolitan Government and were separate programs from one another. Out of 55 randomly selected programs, two programs were unable to participate in the survey. Program participants totaled 1,152 persons. Program administrators of the remaining 53 CRPs and the primary caregivers of the adults participating in the programs were also identified. Inclusion criteria for the study's sample were based on whether the participant has been involved in the program for at least three months or more than ten times; was above 40 years old at the time of the study; and provided information on gender. Based on these criteria, 22 participants were identified as younger than age 40 and one participant was missing information on gender. Hence, the final sample comprised of 1,129 program participants, or 480 females and 649 males.

Data collection was based on a survey method distributed and collected in person by employees of the respective CRPs. Questionnaires were taken from the program administrators and primary caregivers, respectively. Then, the questionnaires were collected by the employees of the respective CRPs. Information was obtained from program administrators about (1) their program and (2) the basic characteristics of the program participants. As for their program characteristics, the administrators were specifically asked about the type of transportation services they offered: pick up at a bus stop, pick up at home by car (hereinafter, door-to-door), or other types of transportation services such as providing taxi vouchers and program-run vehicles. Basic characteristics of program participants were based on the records: gender (categorical variable, 1 = male or 0 = female); age (continuous variable, number of years); and presence of reported causal diseases leading to disability. Two standardized scales were also included. Composite score measuring mental status was based on Hasegawa Dementia Scale (HDS, 0 = dementia to 32.5 = normal) [11]. Functional status was measured as a composite score using a standardized format created by Tokyo Metropolitan government consisting of seven aspects of which six aspects were from Katz' ADL measure [12] and one aspect of total mobility on a three-point Likert-scale (7 = independent to 21 = most dependent). Validation of the ADL measure used in the study was confirmed by comparing with Katz's ordinal scale by the author [13].

A separate questionnaire was conducted to obtain information from the primary caregivers of the program participants on their use of CRPs. The survey consisted of five major questions. Questions related to family and family support included (1) his/her relationship to the program participant as measured by the relationship of the caregiver and the program participant (categorical variable), (2) number of family members living with the participant (continuous variable, number of family members), and (3) whether the primary caregiver accompanied the participant to a CRP (categorical variable). The question on (4) the use of the program was measured by the frequency of program attendance during the last three months (summative score). They were also asked about (5) the use of other health services including rehabilitation at the hospital, visit to a primary physician, and whether the participant had obtained a pass for accessing welfare services. Informed consent was obtained from participants prior to their involvement in the study.

Responses from the questionnaires were analyzed using PC-SAS Version 8. Bivariate statistical analyses were conducted using Chi-square tests for categorical variables and *t*-test or ANOVA for continuous variables, respectively.

## Results

### Basic characteristics of the program participants

Characteristics of the program participants were summarized in Table 1. In the year which the data was collected for this study, the estimated total population of the community-based older adults with disabilities in Tokyo was 114,087 (43%) males and 150,402 (57%) females [14]. With adjustment in our final sampling rate: 24% (programs selected/total number of programs in Tokyo, or 53/220), estimated population at risk was 27,381 males and 36,096 females. Therefore, the approximate participation rates for CRPs for our study sample were estimated as 2.3% (649/27,381) for males and 1.3% (480/36,096) for females. If our assumption is true, the participation rate of females was significantly less than that of males (*X*^2 ^= 93.0, p < 0.0001).

**Table 1:** Characteristics of community rehabilitation program (CRP) participants by gender (n = 1,129)

	**Male mean ± *SD *n (%)**	**Female mean ± *SD *n (%)**	***X***^2^	***P***
***N***	649	480		
**Demographics**				
Age	67.2 ± 9.4	69.4 ± 10.8		.003^1)^
Number of times attended CRP in the past 3 months	17.7 ± 11.6	16.0 ± 10.4		.015^1)^
**Disability and disease**				
Difficulty with activities of daily living (ADLs) (7 = independent to 21 = most dependent)	9.6 ± 3.2	9.0 ± 3.0		.006^1)^
Dementia (0 = normal to 7 = dementia)	0.2 ± 0.8	0.2 ± 0.9		.553
Cardiovascular disease (CVD)	532 (82%)	286 (60%)		< .0001^2)^
Hemiplesia	469 (72%)	260 (54%)	69.3	< .0001^2)^
Proportion of Hemiplesia in CVD	469/532(88%)	260/286(93%)	39.5	
Duration from onset	6.9 ± 7.7	8.7 ± 10.4		.001^1)^
**Family Support**				
Spouse as primary caregiver	465 (72%)	174 (36%)	140.8	< .0001^2)^
Accompanied by caregiver to the program	243 (37%)	146 (30%)	6.03	.014
**Type of referrals to CRP**				
Referred by a friend	85 (13%)	113 (24%)	20.8	< .0001^2)^
Referred by a hospital	156 (24%)	90 (19%)	4.53	.033^2)^
Through newspapers	119 (18%)	61 (13%)	6.52	.01^2)^
**Use of other services**				
Hospital rehabilitation program	525 (81%)	318 (66%)	31.3	< .0001^2)^
Primary physician	635 (98%)	461 (96%)	3.2	.076
Social service pass	537 (83%)	341 (71%)	21.9	< .0001^2)^

^1) ^*t*-test

^2) ^Chi-square test (2 × 2)

Note: The number of respondents (as denominator) is different in each item.

Female participants were found to be older than male participants (females, M = 69.4, *SD *= 10.8; males, M = 67.2, *SD *= 9.4; *p *= 0.03), and experienced less difficulty with ADLs compared to males (females, M = 9.6, *SD *= 3.2; males, M = 9.0, *SD *= 3.0; *p *= .006). As for causal disease leading to disability, cardiovascular disease (*p *< 0.001) and hemiplesia (*p *< 0.001) were found to be more frequent in males than females. The duration since the onset of the disease, however, was longer for females than males (*p *< 0.001). In terms of disease comorbidity, a majority of participants with CVD had hemiplesia (females 93%; males, 88%).

As for the relationships with caregivers, there were more male participants than female participants with spousal caregivers (72% and 36%, respectively; *p *< 0.001). Number of family members living together did not differ statistically by gender.

When asked how they found out about CRPs, more female participants reported that they learned about the service through a friend (*p *< 0.001), while more males found out about CRPs through hospital referrals (*p *= 0.033) or newspapers (*p *= 0.01). As for other health service use, more males than females reported prior use of hospital rehabilitation programs, and had obtained a certified pass for social welfare services (*p *< 0.001).

**Figure 2:**
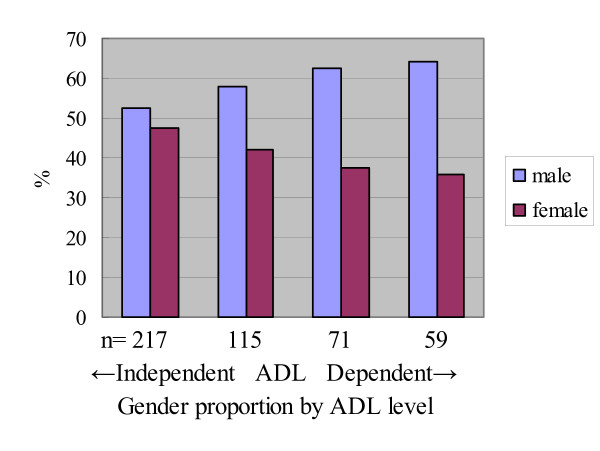
**Gender proportion and ADL levels of all participants (n = 1,085)**.

Participants' ADL and gender proportions were then stratified by accompanying status of caregivers and transportation services in Tables 2 and 3. Since the proportion of females tended to be smaller than males when ADL dependence increased (Figure 2), we assumed that this difference in ADLs by gender is due to the gender differences in availability of transportation services and accompanying caregivers (Table 1).

**Table 2:** Comparison of gender and ADL of participants by accompanying status (n = 1,085)*

**Status**	**Participant**	**Gender**			**ADL (mean ± *SD*)**				
	**N (%)**	**Male**	**Female**	***p***	**Total**	***p***	**Male**	**Female**	***p***

Accompanied	372	231 (37%)	141 (31%)		10.9 ± 3.6		11.4 ± 3.6	10.9 ± 3.5	.174

Unaccompanied	713	395 (63%)	321 (69%)	*p *= .014	8.18 ± 2.4	*p *< .0001	8.7 ± 2.4	8.5 ± 2.3	.157

Total	1085*	623 (100%)	462 (100%)				9.3 ± 3.2	8.8 ± 3.0	.006

* missing data on ADLs (n = 44)

**Table 3:** Comparison of gender and ADL of participants (n = 1,085)* by different types of transportation services offered by programs (n = 53)

**Service**	**Programs**	**Participant**	**Gender**			**ADL (mean ± *SD*)**				
	**N (%)**	**N (%)**	**Male**	**Female**		**Total**		**Male**	**Female**	***p***

No service	20 (38%)	215 (20%)	138(64%)^a^	77 (36%)		8.8 ± 3.0		9.7 ± 3.3	8.8 ± 2.4	.023

Bus stop	13 (25%)	395 (36%)	215 (54%)	180 (45%)		9.0 ± 2.8		9.7 ± 3.0	8.9 ± 2.5	.005

Door-to-door	17 (32%)	411 (38%)	224 (55%)	187 (46%)	*p *= .007^b^	9.9 ± 3.4	< .0001^c^	10.5 ± 3.5	10.3 ± 3.4	.482

Other	3 (6%)	64 (3%)	46 (72%)	18 (28%)		9.7 ± 3.0		9.7 ± 3.0	9.6 ± 3.0	.941

Total	53 (100%)	1,085 (100%)	623 (57%)	462 (43%)		9.3 ± 3.1		9.3 ± 3.2	8.8 ± 3.0	.006

* missing data of ADL (n = 44)

^a ^Total number in each service = 100%

^b^Chi-Square test (2 × 4)

^c^ANOVA

### Caregiver's accompanying status

Table 2 summarized the results of whether program participants were accompanied by their caregivers to CRPs. Participants accompanied by primary caregivers accounted for 37% of males, which was significantly higher than females (31%, *p *= 0.014). ADL levels of accompanied participants were significantly more dependent (*p *< 0.001). Gender difference in ADL levels for all participants (p = 0.006) were no longer statistically significant when stratified by accompanying status.

### Use of transportation services

Table 3 showed results on gender differences by transportation services provided by CRPs. Twenty program administrators reported that their programs don't offer any transportation services, while 33 administrators reported offering one type of transportation service at each of their programs. Thirteen programs offered bus service by picking up participants at their nearest bus stops. Seventeen programs provided door-to-door services. Three programs provided other transportation services such as taxi vouchers subsidized by the program.

Gender proportion and ADL level compared by types of transportation services offered by CRPs were significantly different (*p *= 0.007 and *p *< 0.001, respectively). The proportion of female participants in the CRPs with door-to-door transportation service was highest among all the categories. When comparing ADL levels by the types of transportation service, significant differences were found. Participants of CRPs with door-to-door transportation service were the most ADL dependent (ADL score: 9.9+3.4). Gender differences in ADL were no longer found in CRP participants with door-to-door services (males 10.5+3.5; females 10.3+3.4), or other types of transportation service (males 9.7+3; females 9.6+3.0). However, there were significant differences in the total population (males 9.3+3.2; females 8.8+3.0). Gender difference was found among participants at CRPs with no transportation services and CRPs with bus service.

### Gender proportions in different ADL levels stratified by transportation services

**Figure 3:**
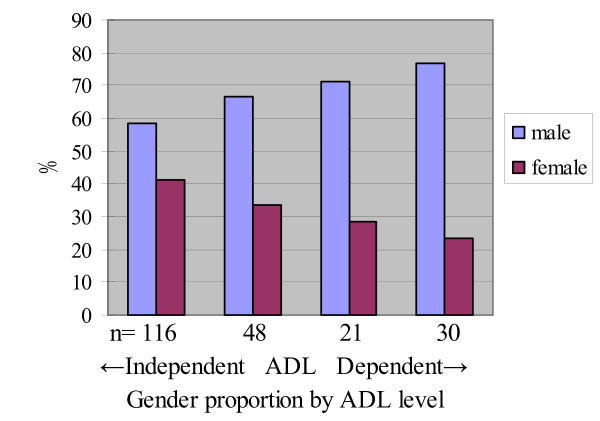
**Gender proportion and ADL levels of no transportation services group (n = 215)**.

**Figure 4:**
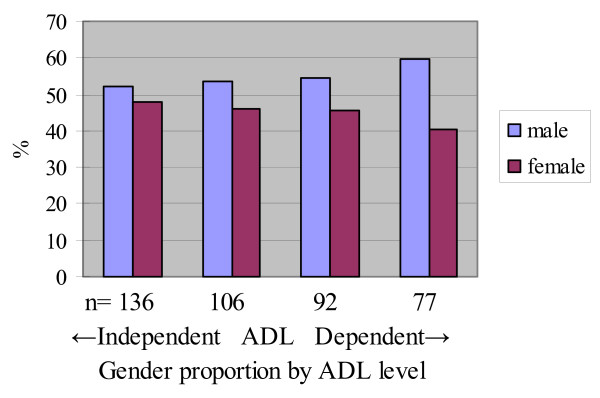
**Gender proportion and ADL levels of door-to-door transportation services group (n = 411)**.

**Figure 5:**
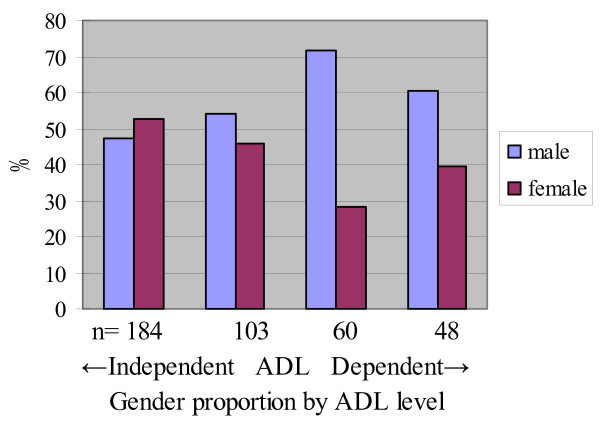
**Gender proportion and ADL levels of bus stop pickup transportation service group (n = 395)**.

Gender proportions by ADL level in quartiles were stratified by transportation services as shown in Figures 3 and 4. Difference in gender was larger in the group of participants attending CRPs with no transportation services (Figure 3). In contrast, the gender differences by ADL dependence were smaller in the group with door-to-door transportation services (Figure 4) and for bus stop pickup transportation service (Figure 5).

## Discussion

Our study underscores two findings: (1) there is gender difference in utilization of community rehabilitation programs in Tokyo, and (2) lower use of CRPs by females living in Tokyo is related to the unavailability of transportation services and accompanying persons to the nearest transportation site. In other words, the study suggests that prospective CRP participants were not able to attend the program without family members accompanying them from home to the bus stop or without the provision of comprehensive transportation services by the CRPs. It is likely that removing these barriers will decrease the gender difference in use of CRPs.

In addition to the lack of available transportation services for females, our findings suggest that the lack of available transportation service for male participants could be solved by having spousal caregivers accompany their family members. We assume that the overall effects of transportation by gender are more pronounced by the social expectations placed upon family caregivers to take an active role in caring for their older adults. Traditionally, Japanese society has a reputation for providing traditional family care for older family members. Although the percentage of Japanese reporting filial care as a natural responsibility has declined over the past 40 years, a report published in 2006 shows that approximately half of the Japanese persons continue to think positively that familial care for older adults is a cultural norm:30%, or good practice:20% [15]. It has also been reported that 57.8% of older adults showed a preference for spouses as caregivers followed by daughters or daughters-in-law (24.5%) [16]. Hence, largely influenced by the cultural preference for familial care of older adults, Japan's policy for older adult's care still remain heavily dependent on the family. However, there may be a growing gap of understanding between social expectations and actual caregiving realities. Moreover, the situation may have altered with the implementation of the national long-term care insurance. Therefore, future research on the use of transportation services under the new policy climate is recommended.

Reasons for attending CRPs also differed by gender. Males were introduced to CRPs by hospital or newspapers while females found out about the programs through friends. Based on their study on use of rehabilitation services, Altman and Smith reported that males were more likely than females to be placed in rehabilitation programs based on medical model than psychosocial model [17]. Higher percentage of males using hospital rehabilitation may be caused by the higher prevalence of CVD among males, but also related to male dominance in the medical model.

Whether these findings speak to specific preferences of services by gender or not, our study's findings suggest that gender remains as a serious consideration for organizing and planning interventions for improving access to health care for older adults. Setting aside government budget targeted specifically to fund vehicles and drivers equipped to carry passengers with disabilities, or relieving the transportation costs for patients are some on the strategies to improve access to community rehabilitation programs. With the implementation of the NLTCI in 2000, transportation to rehabilitation programs were supported by the government through additional reimbursements and thus, access to programs was thought to have improved. However, with the revisions of the NLTCI in 2006, additional reimbursements for transportation services were discontinued. Instead, total reinvestment for the CRP was raised with hopes that existing transportation services will be covered under the revised pay structure. We can only assume that the transportation services provided by the CRPs may have declined with the lack of additional government support. The government has not released any data on the number of transportations services offered or discontinued nor are there any other studies to date examining the impact of transportation services on service use by NLTCI beneficiaries. Based on our findings, any decrease in the number of transportation services due to the discontinuation of reimbursements towards transportation by the NLTCI may impact female patients' access than male patients. Research examining the role of transportation services for accessing CRPS requires further investigation under the revised NLTCI.

Other solutions to solve inaccessibility to health care services are to innovate new means for access, and/or duplicating similar services across the health care continuum. An alternative recommendation reflective of these solutions is to offer a holistic service at CRPs not limited to offering rehabilitation for older participants, but also to include services for caregivers. This may motivate caregivers to accompany their older family members to CRP. It joins both caregiver-focused program and rehabilitation program as one, and allows overlapping of resources.

This study has three limitations. First, our data only had the estimated participation rates by gender, and did not have the actual rates. Therefore, it is unclear whether there was gender difference in the real need for the CRPs. Second, we also do not know whether the benefits of participating in CRPs reaped different results by gender. In general, we do know that females were more likely to visit a doctor than males [18], so we hypothesize that a lack of motivation to use CRPs wasn't a factor. Finally, our findings are limited for generalizablity due to the nature of retrospective data. Since our study examined only older adults who were already participating in CRPs, our findings may be skewed. In fact, given the long life expectancy rate of the Japanese, our sample was relatively young and the level of disability rate was also relatively low. However, we believe our study was able to deliver the message that Japanese females living in Tokyo were less likely to access CRPs although they were older with higher levels of disability, and residing in the community.

## Conclusion

In conclusion, this study builds on previous research findings, which suggest gender inequality in access to CRP. While this analysis may help explain gender differences in the use of CRPs in Japan, this study only serves as one step towards understanding this difference. Unfortunately, very little information exists on the relationship between older adult's mobility and transportation [19]. Moreover, gender difference in health services for older adults has not been paid much attention in Japan. Therefore, this study has contributed to the limited number of research investigating the role of transportation as a structural barrier within the health care system. Prospective studies will further this area of study to grasp a better picture of older adults' needs for and access to CRPs.

## Competing interests

The authors declare that they have no competing interests.

## Authors' contributions

NT obtained the mandate to perform the study, performed statistical analyses, and wrote the draft of the manuscript. LC reviewed the literature and wrote the subsequent versions of the manuscript with NT. YK contributed to the research design and conducted statistical analyses of the data with NT. MK organized and oversaw the entire data collection process. EY supervised the entire study and provided academic guidance throughout the study process. All authors read several versions of the manuscript, provided input to the intellectual content, and approved the final manuscript.

## Pre-publication history

The pre-publication history for this paper can be accessed here:


